# Phenotypic Variance Predicts Symbiont Population Densities in Corals: A Modeling Approach

**DOI:** 10.1371/journal.pone.0009185

**Published:** 2010-02-12

**Authors:** Robert van Woesik, Kazuyo Shiroma, Semen Koksal

**Affiliations:** 1 Department of Biological Sciences, Florida Institute of Technology, Melbourne, Florida, United States of America; 2 Department of Mathematical Sciences, Florida Institute of Technology, Melbourne, Florida, United States of America; University of Hull, United Kingdom

## Abstract

**Background:**

We test whether the phenotypic variance of symbionts (*Symbiodinium*) in corals is closely related with the capacity of corals to acclimatize to increasing seawater temperatures. Moreover, we assess whether more specialist symbionts will increase within coral hosts under ocean warming. The present study is only applicable to those corals that naturally have the capacity to support more than one type of *Symbiodinium* within the lifetime of a colony; for example, *Montastraea annularis* and *Montastraea faveolata.*

**Methodology/Principal Findings:**

The population dynamics of competing *Symbiodinium* symbiont populations were projected through time in coral hosts using a novel, discrete time optimal–resource model. Models were run for two Atlantic Ocean localities. Four symbiont populations, with different environmental optima and phenotypic variances, were modeled to grow, divide, and compete in the corals under seasonal fluctuations in solar insolation and seawater temperature. Elevated seawater temperatures were input into the model 1.5°C above the seasonal summer average, and the symbiont population response was observed for each location. The models showed dynamic fluctuations in *Symbiodinium* populations densities within corals. Population density predictions for Lee Stocking Island, the Bahamas, where temperatures were relatively homogenous throughout the year, showed a dominance of both type 2, with high phenotypic variance, and type 1, a high-temperature and high-insolation specialist. Whereas the densities of *Symbiodinium* types 3 and 4, a high-temperature, low-insolation specialist, and a high-temperature, low-insolation generalist, remained consistently low. Predictions for Key Largo, Florida, where environmental conditions were more seasonally variable, showed the coexistence of generalists (types 2 and 4) and low densities of specialists (types 1 and 3). When elevated temperatures were input into the model, population densities in corals at Lee Stocking Island showed an emergence of high-temperature specialists. However, even under high temperatures, corals in the Florida Keys were dominated by generalists.

**Conclusions/Significance:**

Predictions at higher seawater temperatures showed endogenous shuffling and an emergence of the high-temperature *Symbiodinium* specialists, even though their phenotypic variance was low. The model shows that sustaining these “hidden” specialists becomes advantageous under thermal stress conditions, and shuffling symbionts may increase the corals' capacity to acclimatize but not adapt to climatechange–induced ocean warming.

## Introduction

The ubiquity of modern reef-building corals in the shallow, low-nutrient tropical environments stems from their capacity to house unicellular dinoflagellates [1]. This mutually beneficial relationship depends on photosynthates that are released by the symbionts and utilized by the coral host; corals, in turn, produce organic wastes upon which the symbionts thrive [2], [3]. Coral symbionts, or *Symbiodinium* species, were once thought to consist of only one species [4]. However technological advances show potentially hundreds of symbiont types [5]–[9], and some preliminary research has shown that coral physiology is highly dependent on the type of symbionts present in the host [10]–[13].

Most corals seem very specific in the type of *Symbiodinium* they support, and most corals only support one *Symbiodinium* type over time [14]–[16]. Still, some coral species are capable of simultaneously supporting more than one *Symbiodinium* population, which are spread across coral colonies in accordance with down-welling irradiance [17], [18]. *Symbiodinium* population densities are not, however, in a steady state. Population densities vary in accordance with seasonal temperature, irradiance and nutrient concentrations [19]–[24]. Recently, Chen et al. [23] demonstrated seasonal dynamics in the relative densities of different *Symbiodinium* types, presumably upregulating the high-light, high-heat tolerant species in summer. Several key studies have also shown seasonal declines in photosynthetic efficiency that is related to high seawater temperature and irradiance [25], [26]. Corals pale, or bleach, when temperatures exceed seasonal averages for extended periods [27]. An extreme case-in-point is the 1997–98 global thermal stress event, which was an extreme manifestation of a more general impact of the El Niño-Southern Oscillation cycle. This event led to extreme coral bleaching and extensive coral mortality worldwide [28].

Symbiotic scleractinian corals live close to their thermal tolerance levels. The last two decades have seen an increase in the frequency and severity of symbiotic dysfunction (i.e., coral bleaching) in response to anomalous sea-surface temperature increases [29]–[33]. Yet, symbiont responses vary in accordance with the type of stress [3]. If temperature and irradiance stresses are of moderate intensity and duration, corals are capable of regaining pigmentation, both through increases in *Symbiodinium* pigment and population densities [34]. If stress exceeds a critical threshold, which varies among coral species and geographic locality [35], [36], [10], bleaching is inevitable, often leading to partial or whole-colony mortality [37]–[39].

Contemporary molecular-ecology research is interested in the dynamics of *Symbiodinium* in corals, their response to thermal stress events [40]–[42], and what role the *Symbiodinium* might play in acclimatization and adaptation of reef corals [43]. We note that the present study is only applicable to those corals, approximately 25% of corals worldwide [16], that naturally have the capacity to support more than one type of *Symbiodinium* within the lifetime of a colony, for example *Montastraea annularis* and *Montastraea faveolata.*

### Models

Ware et al. [44] devised a mathematical model to examine *Symbiodinium* population growth during and after thermal stress events using generalized Lotka-Volterra competition equations. Although Ware's model predicts the superior *Symbiodinium* type, the system is set such that the differential equation that governs *Symbiodinium* type 1 (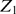), the first equation in the set, will ultimately dominate the entire system. The model does not consider resources for which *Symbiodinium* species compete. We sought to examine the response of *Symbiodinium* population densities to the seasonal dynamics of solar insolation (a resource) and seawater temperature.

Recent research on adaptation to climate change and increasing thermal stresses has emphasized the need to assess phenotypic variance of organisms in general [45], [46] and corals in particular [43], [47]. We test whether the phenotypic variance of symbionts may be closely related with the capacity of *Montastraea* corals to acclimatize to increasing seawater temperatures. Moreover, we assess whether more specialist symbionts are lost from the coral (holobiont) under a warming ocean. The objectives are to obtain accurate time-course predictions of *Symbiodinium* population densities in *Montastraea* corals, and make valid estimates, of *Symbiodinium* densities, under seasonal dynamics of solar insolation and seawater temperature, and through thermal stress events.

## Materials and Methods

### Symbiont-Population Growth

Growth of each *Symbiodinium* population can be modeled by considering specific growth rates relative to specific loss rates. Population flux can be theoretically estimated (following Jones and Yellowlees [48]) using the difference equation:



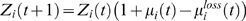
(1)where 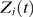 is the population density of *Symbiodinium* (or zooxanthellae) type *i*, 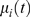 is the specific growth rate of 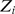, and 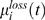 is the specific loss rate of 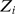 from the host coral at time 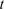 (Table 1). An assumption of the model is that the resources allocated to each *Symbiodinium* population influences 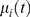, and that down-welling solar insolation is the primary resource limiting symbiont population densities (see Table 2 for other assumptions). We note that high insolation, in early summer, leads to photoinhibition and reductions in symbiont population densities. Furthermore, increasing nutrients can have the opposite effect of increasing symbiont densities [49], [22], [14]. But nutrient concentrations are far less predictable than insolation and temperature [50], and are therefore not input into our model. Where nutrient concentrations (X) are available, then X can be defined as a function of time f(t), and inserted as a resource in Equation 3 (below).

**Table 1:** Notations and abbreviations used in the optimal-resource model.

Notation	Unit	Interpretation
*t*	day	time
Z	cells cm^−2^	zooxanthellae density
*μ_i_*	dimensionless	proliferation rate of zooxanthella type *i*
*μ^loss^*	dimensionless	zooxanthellae loss rate from host
r*_pro_*	kWm^−2^d^−1^	resource for zooxanthellae proliferation
R*i*	resource dependent	required resource for proliferation of zooxanthella type *i*
*a, b, c*	dimensionless	coefficients of environmental parameters [51]
*SI*	kWm^−2^d^−1^	solar insolation
*SI_i_^opt^*	kWm^−2^d^−1^	optimal *SI* for zooxanthella type *i*
*hSI^opt^*	kWm^−2^d^−1^	optimal *SI* for host
*SST*	°C	sea surface temperature
*SST_i_^opt^*	°C	optimal *SST* for zooxanthella type *i*
*hSST^opt^*	°C	optimal *SST* for host
*α*	kWm^−2^d^−1^	optimal *SI* range for zooxanthella type *i*
*β*	°C	optimal *SST* range for zooxanthella type *i*
γ	kWm^−2^d^−1^	optimal *SI* range for host
*K*	cells cm^−2^	carrying capacity - host dependent
*Kc*	cells cm^−2^	environmentally dependent carrying capacity
∑ *Zi*	cells cm^−2^	total number of zooxanthellae cm^−2^

**Table 2:** Assumptions used in the optimal-resource model.

1)	Host corals may possess multiple *Symbiodinium* types at any given time and exogenous *Symbiodinium* do not contribute to any population densities;
2)	*Symbiodinium* proliferation rate is driven by the dynamic resource solar insolation;
3)	Solar insolation and seawater and temperature covary [66];
4)	*Symbiodinium* density is a balance between specific growth and loss rates;
5)	The growth response function of each *Symbiodinium* type follows a Gaussian distribution [52].

**Figure 1:**
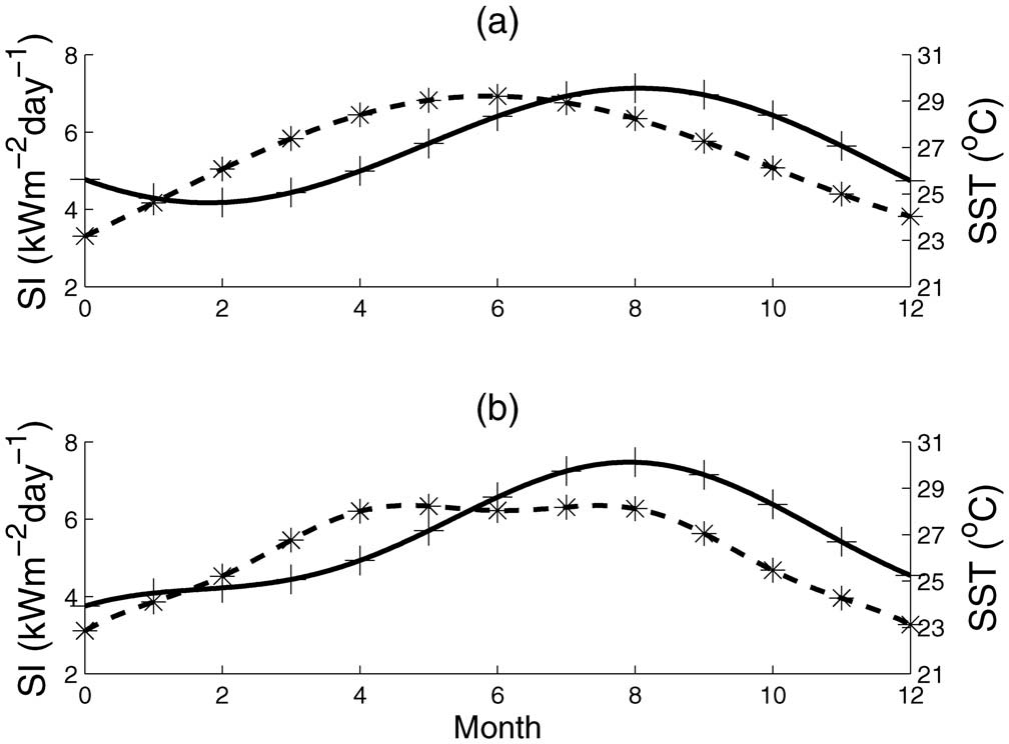
Dynamics of 10-year seasonal means of Sea Surface Temperature (SST) (solid line) and Solar Insolation (SI) (dashed line). Panel (a) shows the dynamics for Lee Stocking Island, the Bahamas, and panel (b) shows the dynamics for Key Largo, Florida.

*Symbiodinium* population densities were predicted for corals at Lee Stocking Island (23°N, 76°W) and Key Largo (24°N, 80°W) using solar insolation (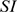) (kW m^−2^ d^−1^) as a primary resource at each location using the general equation:



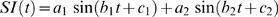
(2)where 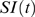 is solar insolation at time *t*, and *a_i_*, *b_i_*, and 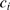 were locality specific coefficients, while temperature is not a resource, but rather a condition. From satellite data [51], ten-year averages of solar insolation and sea surface temperature were used to derive functions with respect to time (Figure 1). For simplicity, annual change in Sea Surface Temperature (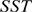
in °C) followed the same general construct, replacing 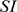 with 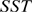
in Equation 2 and the parameters were changed appropriately for each location.

### Competition for Resources

An average *Symbiodinium* is generally no more than 10 µm diameter, and 10^6^ *Symbiodinium* cells can fit in 1 cm^2^ of coral tissue depending on tissue thickness, which can vary from 0.3 to 10 mm depending on the coral species under examination. Deeper *Symbiodinium* receive less light than surface *Symbiodinium*. Since solar insolation is the primary resource considered here, the resource becomes limiting with an increase in *Symbiodinium* density. Therefore, *Symbiodinium* proliferation rate, 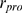, (following Tilman et al. 1997 [52]) can be described as a function of time:



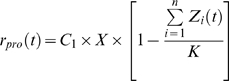
(3)where 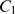 is a constant coefficient; 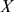 is the primary resource (here solar insolation (*t*)); 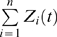 is the total number of *Symbiodinium* cm^−2^; and 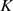 is the carrying capacity within the host corals. While this model examines changes in symbiont dynamics over time, it is equally appropriate to examine micro-environmental profiles, such as those reported in Rowan et al. [17]. Partitioning coral colonies into different micro-irradiance environments, for example, would be equally valid.

**Figure 2:**
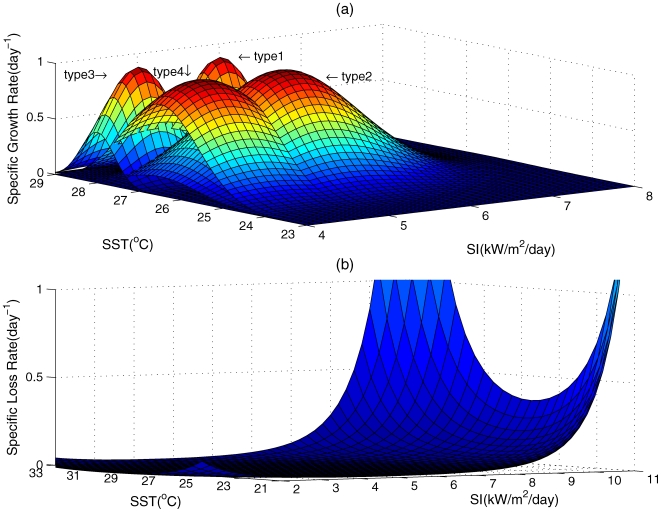
*Symbiodinium* growth and loss rate response curves in relation to Sea Surface Temperature (SST) and Solar Insolation (SI). Panel (a) shows specific growth rates of four *Symbiodinium* types, and panel (b) shows specific loss rates.

The specific growth rate, 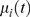, of *Symbiodinium* 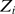 is given as:



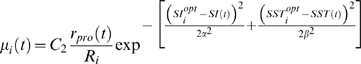
(4)where 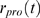 is the resource allocation to *Symbiodinium* proliferation at time 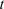 (derived in Equation 3); 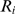 is the resource requirement for 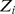; 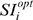
and 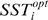 are optimal proliferation requirements (following Pulliam 2000 [53]) of 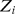 with regard to solar insolation (*SI*) and sea surface temperature (*SST*); 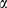 and 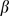 are standard deviations of *SI* and *SST* (Figure 2a); and *C_2_* is a constant coefficient. For each *Z_i_*, 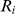 was set to 1, with all *Symbiodinium* showing equal competitive abilities for resources. If physiological studies find otherwise, R_i_ can be set hierarchically, with the most competitive *Symbiodinium* type set at *i* = 1, and the most inferior type set at *i* = *n*. Dynamics of the sustainable *Symbiodinium* density for each Z*_i_* in host corals are expressed by:



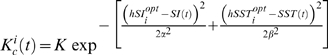
(5)


Because no data are available to the contrary, excess symbionts relative to 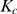, are assumed to be lost from the host corals randomly and independent of *Symbiodinium* type. The specific *Symbiodinium* loss rate is:



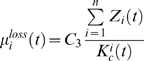
(6)where *C_3_* is a constant coefficient (Figure 2b).

### Population-Density Predictions

To predict *Symbiodinium* population densities, the following conditions were applied to the models:

1. values of 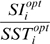 were 5.5/28, 5.5/26.5, 4.5/28, and 4.5/26.5, for each 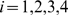, respectively, which covers the range of solar insolation and seawater temperature probabilities for the Florida Keys and the Bahamas (using the units °C for sea surface temperature and kW m^−2^ d^−1^ for solar insolation) (Figure 2);
2. the standard deviations for 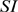 were 0.4, 0.8, 0.4 and 0.8 for each 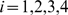, respectively (Figure 2) – we define *Symbiodinium* types 1 and 3 as specialists because they have narrow environmental tolerances;
3. the standard deviations for 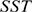
were 0.4, 1, 0.4 and 1 for each 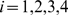, respectively – again, *Symbiodinium* types 1 and 3 are defined as specialists because they have narrow environmental tolerances;

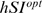
 and 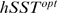 for the holobionts, were 5.5 and 27 for SI and SST respectively;
5. standard deviations for 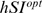
and 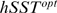
were 2.0 and 3.0, respectively;
6. each *Symbiodinium* type had an initial population density of 1.0×10^6^ cells cm^−2^;
7. each month was set at 30 days, and one year was set at 360 days;

Since Equation 1 is a discrete time model, the solutions (i.e., population densities) were approximated in discrete time (10 yr) by numerical iteration. The SI_i_^opt^ and SST_i_^opt^ (values for condition 1 above) and the values for the standard deviations (for conditions 2 and 3 above), were derived from normal distributions for each zooxanthellae type for *each* iteration step (with mean, μ, and the standard deviation, σ, of the distributions given in conditions 1 and 2, respectively). To introduce real-world thermal stress, *Symbiodinium* populations were randomly subjected to +1°C above-average temperatures in July, +1.5°C in August, and +1°C in September. The results were compared with a 4-year study, which tagged host corals and regularly monitored *Symbiodinium* types and their densities from 2000 to 2004, in Key Largo, Florida, and Lee Stocking Island, the Bahamas [41].

## Results and Discussion

### Seasonal Dynamics

**Figure 3:**
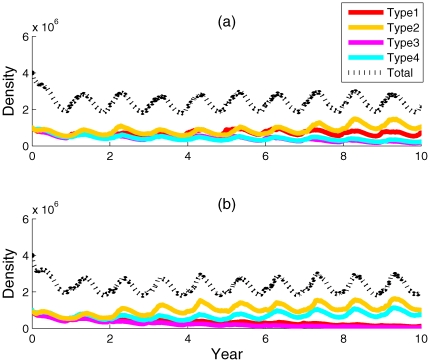
Ten-year iterations of four *Symbiodinium* population densities in corals modeled at two Caribbean localities. The models were run with C_1_ = 0.01, and R_1_ =  R_2_ = R_3_ = R_4_ = 1. Panel (a) shows the results for Lee Stocking Island, the Bahamas and panel (b) shows the results for Key Largo, Florida.

**Figure 4:**
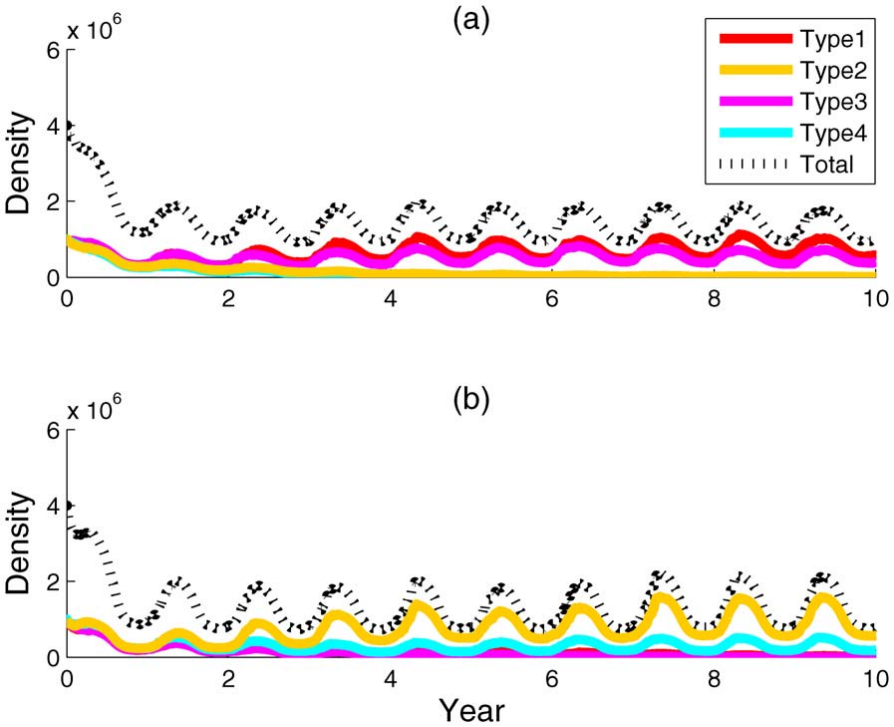
Ten-year iterations of four *Symbiodinium* population densities in corals that have been subjected to above-average water temperature increases. Where panel (a) represents predictions for Lee Stocking Island, the Bahamas, subjected to above-average temperatures of +1°C July, +1.5°C August, +1°C September; and panel (b) represents predictions for Key Largo, Florida, subjected to the same above average temperatures.

*Symbiodinium* densities varied seasonally, showing highest densities from December to April; extreme solar insolation and temperature conditions induced high 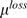 in summer for both localities (Figures 2, 3). *Symbiodinium* dynamics were more variable in Key Largo, Florida, than at Lee Stocking Island, the Bahamas (Figure 3). Predictions for Lee Stocking Island, the Bahamas, where temperatures were relatively homogenous throughout the year, showed a dominance of *Symbiodinium* type 2, which had high phenotypic variance, and a type 1 high-temperature and high-insolation specialist (type 1). The densities of *Symbiodinium* types 3 and 4 remained consistently low (Figure 3). In contrast, predictions for Key Largo, Florida, where environmental conditions were more seasonally variable, showed the co-dominance of two *Symbiodinium* populations (types 2 and 4), both with high phenotypic variance. The specialist symbionts, types 1 and 3, with low phenotypic variance, were present but in very low densities (Figure 3). At elevated temperatures, population densities showed endogeneous ‘shuffling’ at both sites and an emergence of types 1 and 3, the high-temperature specialists, with low phenotypic variance, at Lee Stocking Island (Figure 4). In contrast, the elevated temperatures allowed types 2 and, somewhat less of, type 4 to remain dominate in Key Largo corals, with extremely low densities of types 1 and 3 specialists (Figure 4).

In 2006, Thornhill et al. [41] noted that *Symbiodinium* in *Montastrea annularis* and *Montastrea faveolata* varied in accordance with locality and depth. They also showed a 2–3 year changeover from one symbiont to another in certain shallow colonies from Florida, and that *M. annularis* and *M. faveolata* supported more *Symbiodinium* types in Key Largo than the same hosts at Lee Stocking Island. Therefore the Thornhill et al. [41] study and the present (modeling) study agree; Key Largo corals support more *Symbiodinium* types than Lee Stocking Island. Thornhill et al. [41] attributed these differences to six potential factors, including environmental variation and human impacts. We suggest insolation and temperature differences between the sites may have the same effect. More interesting, however, was that both studies showed high symbiont diversity directly following extreme thermal stress, followed by stability and reduced diversity. In 2009, Thornhill and colleagues [42] showed that while the 2005 bleaching event caused compositional changes in *Montastrea annularis* and *M. faveolata* symbiont populations, they noted that the recovered genotypes were consistent with the population prior to the thermal stress. Furthermore, they demonstrated remarkable endemism and specificity within host corals. Clearly thermal stress events trigger within-host instability, which may equilibrate through time under more optimal conditions. Still, an increase in the frequency and intensity of disturbance may cause a more ‘permanent’ state of instability.

### Limits

The present model uses a Gaussian distribution to represent environmental optimality. There is no information in the literature confirming or denying such a distribution, although it seems reasonable based on numerous plant-physiology studies [53]. Empirical studies may be best directed at examining physiological variance of *Symbiodinium* in relation to temperature, irradiance and nutrient concentrations. Yet, phenotypic variance may be best expressed as log-normal distributions (i.e., geometric normal) [Gingerich 54]. Similarly, the model assumed random, non-selective *Symbiodinium* loss; selective loss may also follow a Gaussian distribution, but more studies are needed to test this premise. Loss and recovery rates may even follow different distribution functions. For example, loss may follow a continuous exponential or a Weibull distribution, with loss decreasing over time after a threshold is exceeded, while recovery may follow a normal distribution that incorporates a lag-phase. Such adjustments are highly dependent on the outcomes of much needed physiological studies examining *in hospite* responses of *Symbiodinium* to environmental conditions and extremes.

Some studies have clearly shown that *Symbiodinium* population dynamics are influenced by nutrient concentrations [21]. Incorporating nutrient dynamics (in the water column) into the model will require a different approach, especially considering the volatility of many nutrient species and their unpredictability in the environment [50]. A more threshold-based response model may be required to reasonably estimate *Symbiodinium* populations with respect to nutrient dynamics. For example, seasonal extremes (i.e., wet and dry seasons), and event-driven nutrient concentrations may be best input as functions of time (in Equation 3). We input optimality at slightly different parameters; however, theoretically, multiple types of symbionts can also coexist in the same niche space, especially in benign environments where there are no differences between intra- and inter-specific competition [55]–[57]. Although Hutchinson [58] and Huston [59] argued for enhanced diversity at environmentally dynamic localities, because competitive displacement is prevented, the present model predicts that several *Symbiodinium* populations are likely to be present in locations where the physical environment is benign. We add that diversity depends on the phenotypic variance of the populations and highly dynamic localities are less likely to support specialist *Symbiodinium* types.

### Adjustment Capacity

None of the *Symbiodinium* types 1–4 reached zero densities after 10 years, although some densities were extremely low (<1 cell cm^−2^), well below *in situ* levels of detectability (∼5%, which was the state-of-the-art in 2005) [15], [42]. The model showed possibilities of potentially endogenous shifts in the relative abundance of *Symbiodinium* populations, especially under thermal stress. This hidden, vestigial component may be non-adaptive but could become useful when conditions change, especially on reefs away from large land masses. Field studies show that survival through a thermal-stress event, of the multi-claded *Stylophora pistillata* on the Great Barrier Reef, is directly related to whether hosts harbor resistant symbionts [63]. Therefore, sustaining these ‘hidden’ specialists becomes advantageous under thermal stress conditions because the coral holobiont is pre-adapted to thermal stress. In other words, corals harboring multiple symbionts may a have a greater capacity to acclimate to environmental change, but only if those symbionts include thermally tolerant types. Corals harboring thermally sensitive symbionts are rapidly selected out of the gene pool through elevated temperature anomalies [63]. This contrasts with the suggested need to derive novel symbionts from the environment, implied by the adaptive-bleaching hypothesis [60]–[62].

But acclimation reaches a ‘dead end’ under extreme environmental stress; populations can only adjust by evolving – or adapting to the new environment. In principle, a population can adapt to gradual environmental change depending on the amount of genetic variation within a population. But because evolution is the outcome of the interaction between (i) genetic variation, and (ii) natural selection, the capacity to adapt is often limited by the first step – the capacity of a population to produce enough variation upon which selection can act [63]. The second step, in a rapidly changing environment, is ubiquitous and a natural consequence of selective pressure by the environment [64]. Is it then reasonable to assume that corals supporting multiple-species symbionts would have the genetic material to potentially become more thermally tolerant, conceivably adjusting to rapid climate change scenarios, compared with more extinction prone reef corals that strictly support only one specialist *Symbiodinium* type? No. Certainly the multi-symbiont hosts may have a greater capacity to acclimate, but only if they harbor temperature resistant symbionts [65]. There is no evidence that these multi-symbiont hosts have an advantage in their capacity to adapt. Adaptation requires new material, generated through recombination and mutation. Furthermore, a series of independent molecular studies have shown clear evidence of symbiont endemicity [7], [41], [42], suggesting (i) that new symbiont-coral relationships are unlikely in the short term, and (ii) shuffling symbionts is not a mechanism by which corals can adapt to rapidly warming oceans, but it is a useful acclimation mechanism.
